# Case Report: Successful DSA-guided removal of a fractured PICC catheter from the pulmonary artery in a patient with lung cancer

**DOI:** 10.3389/fonc.2025.1656941

**Published:** 2025-08-27

**Authors:** Jun Lai, Xue Xiao, Chunyan Wen, Yaoyu Zhang, Wenjun Meng, Rujun Zheng

**Affiliations:** ^1^ Department of Biotherapy, Cancer Center, West China Hospital, Sichuan University/West China School of Nursing, Sichuan University, Chengdu, China; ^2^ Department of General Surgery, The General Hospital of Western Theater Command, Chengdu, China; ^3^ Department of Urology, The General Hospital of Western Theater Command, Chengdu, China; ^4^ Department of Pain Management, West China Hospital, Sichuan University, Chengdu, China

**Keywords:** lung cancer, chemotherapy, digital subtraction angiography, peripherally inserted central catheter, case report

## Abstract

**Background:**

Peripherally inserted central catheters (PICCs) are widely used for long-term intravenous therapy, especially in cancer patients. Although generally safe, PICC-related complications such as catheter fracture and migration can pose serious risks.

**Case presentation:**

We report a case of a 41-year-old female with lung adenocarcinoma who developed PICC catheter fracture, with the broken segment migrating into the pulmonary artery during chemotherapy. The patient presented without significant symptoms, and imaging confirmed the intravascular migration of the catheter fragment. After multidisciplinary evaluation, the fractured catheter was successfully removed under digital subtraction angiography (DSA) guidance via a minimally invasive endovascular approach. The patient recovered uneventfully.

**Conclusion:**

This case highlights the rare but serious complication of PICC catheter fracture with subsequent migration into the pulmonary artery during chemotherapy in a patient with lung adenocarcinoma. Prompt identification and multidisciplinary management, including minimally invasive retrieval under DSA guidance, ensured a favorable outcome and avoided major morbidity. The experience underscores the importance of standardized PICC maintenance protocols, comprehensive training of nursing staff, and patient education on self-care to minimize preventable complications. Furthermore, our findings emphasize the need for regular catheter monitoring and timely intervention when abnormalities are detected. Minimally invasive endovascular techniques represent a well-documented, safe and effective alternative to traditional surgical methods for the retrieval of intravascular foreign bodies, with advantages of reduced trauma, faster recovery, and fewer complications. Continued efforts to enhance nursing skills, strengthen follow-up, adopt polyurethane catheters instead of silicone PICC per current guidelines, and promote patient awareness are essential to improve the safety and success rates of PICC utilization in cancer populations requiring long-term intravenous access.

## 1 Introduction

Peripherally inserted central catheters (PICCs) are special catheters with small caliber, thin walls, and light weight, which is widely used in patients undergoing cancer chemotherapy, malnutrition, and those who require long-term intravenous medication (1). They are easy to utilize with low risk. More importantly, PICCs can be left in the body for a long time, generally one to two years, thereby alleviating the pain caused by repeated punctures (2). In most cancer patients, a long chemotherapy cycle brings a lot of inconvenience to nursing, so long-term safe placement of PICCs is very important for them. During chemotherapy, PICCs can minimize the probability of phlebitis caused by long-term chemotherapy, tissue damage or necrosis caused by chemotherapeutic drug extravasation (3). Through PICCs, chemotherapeutic drugs can also be delivered to the body more stably, avoiding local tissue damage caused by drug extravasation.

In general, PICCs are a safe, convenient, and effective infusion method. However, during the PICC placement and maintenance process, some side effects may occur. Although PICCs are cheaper to insert and consume less resources than traditional central venous catheters, they are not without the risk of complications (4). Traditional views approve that PICCs are associated with a lower risk of central venous catheter-associated bloodstream infection but a higher risk of deep vein thrombosis than traditional central venous catheters (5, 6), but more recent evidence suggests comparable thrombosis risks with optimized catheter selection and management (7). Among catheter-related complications, catheter breakage and slipping into the vein is rare but a cause for alarm (8). The broken catheter can become an embolus and drift to the cardiac or pulmonary arterial system, and if not treated promptly or effectively, cardiac arrhythmias may occur (9). Digital subtraction angiography (DSA) is commonly used imaging detection methods in clinical practice. It can be used for conventional X-ray film diagnosis and can also provide clear and distinct image references to determine the direction and terminal positioning of the catheter, despite some risks including catheter fracture during removal, with fragments migrating to important blood vessels like the pulmonary artery, which can lead to serious complications such as vascular embolism or even life-threatening situations (10). In this study, we reported a case of a PICC catheter that broke into the pulmonary artery in a lung adenocarcinoma patient receiving chemotherapy and was successfully removed under the guidance of DSA. Our case highlighted that DSA can provide real-time, high-contrast images of the vascular system, allowing the operator to accurately visualize the location and orientation of the fractured catheter fragment.

## 2 Case presentation

A 41-year-old female had been diagnosed with lung adenocarcinoma for more than three years. She had previously received radiotherapy to both the head and pelvis. Then she came to our hospital (West China Hospital of Sichuan University, Chengdu, China) for anti-tumor treatment. After our oncologist’s communication with the patient, systemic chemotherapy with pemetrexed, carboplatin with bevacizumab was getting ready to proceed. Comprehensive pre-procedure assessments, including routine blood tests and coagulation studies, were all within normal limits, and no contraindications to PICC placement were identified. Then, on May 17, 2022, a PICC was placed under ultrasound guidance using the modified Seldinger technique. All procedures were performed in compliance with the ethical guidelines established by the Ethics Committee of West China Hospital of Sichuan University and the Declaration of Helsinki principles. Informed consent was obtained after the risks and procedure were thoroughly explained to the patient and her family.

**Figure 1:**
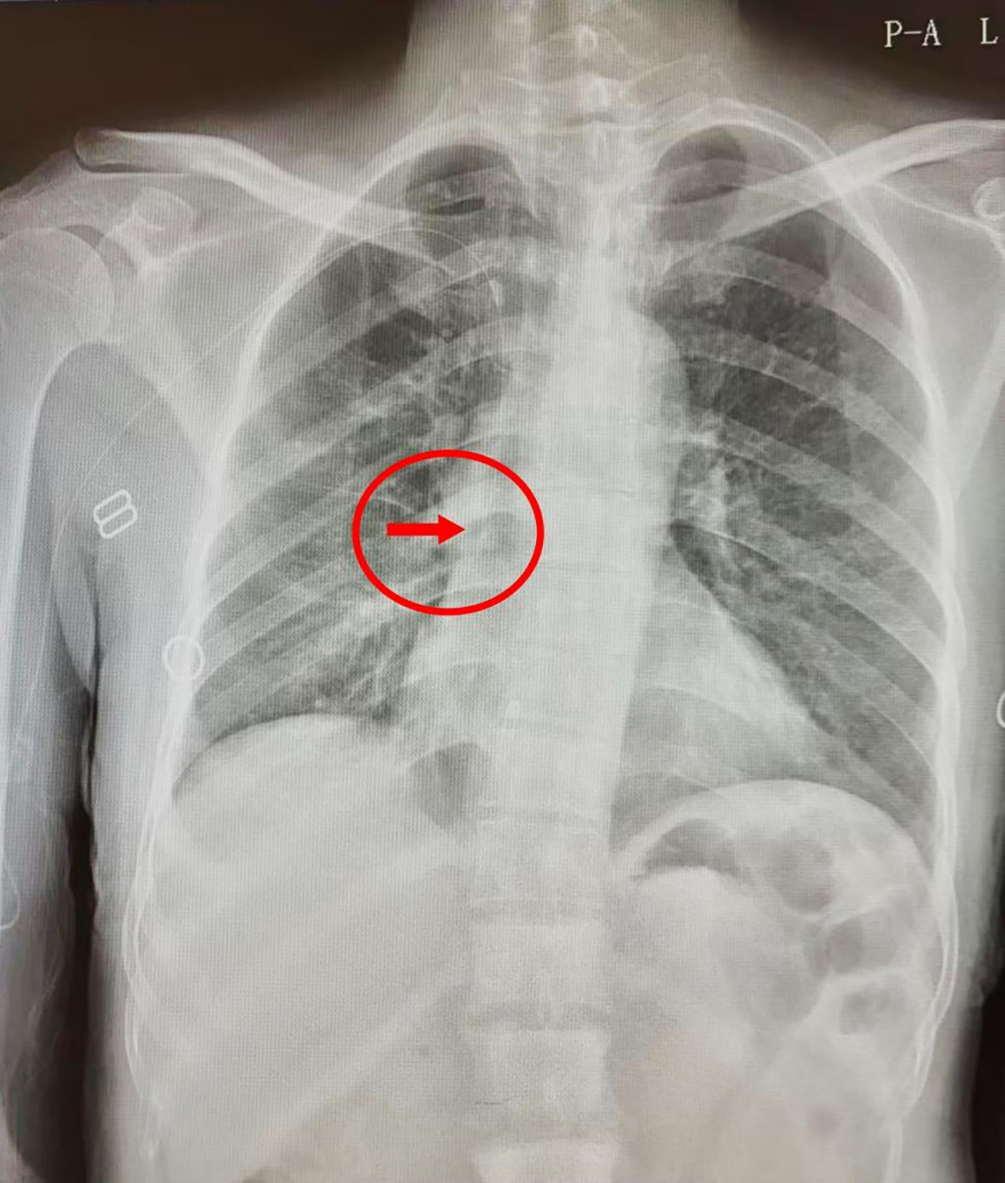
A post-procedural chest X-ray confirmed that the PICC tip was located at the lower edge of the T6/7 vertebra.

The catheterization was performed by a specialist nurse with PICC certification, strictly adhering to standard operating procedures. Under B-ultrasound guidance, a 4F silicone catheter (Groshong catheter, Bard Access Systems Inc., USA) was successfully inserted into the basilic vein of the patient’s right upper arm on the first attempt. The catheter was positioned as planned, with an insertion depth of 36 cm and 4 cm remaining external; the arm circumference measured 27 cm. The patient and her family were provided with detailed instructions regarding catheter care and precautions, which they were able to accurately repeat. A post-procedural chest X-ray was then used for preliminary assessment in our case, though real-time tip location methods (e.g., ECG guidance) are now preferred. The post-procedural chest X-ray confirmed that the catheter tip was located at the lower edge of the T6/7 vertebra (**Figure 1**).

Following PICC insertion, the patient initiated systemic antitumor therapy after careful exclusion of treatment contraindications. The process of PICC implantation ensured safe and effective vascular access for the administration of antitumor therapy.

Beginning 24 hours after catheterization (i.e., from the second day post-insertion), the patient was advised to perform grip exercises using a hand-grip ball with the catheterized limb, completing 100 squeezes per session, three times daily, and continuing this exercise until catheter removal. The recommended technique involved forcefully squeezing the ball, pausing for 1–2 seconds, and then relaxing, in a repetitive manner, which helps minimize the risk of venous thrombosis. To further maintain catheter patency, before and after each infusion, 5–8 mL of physiological saline was flushed through the catheter using a syringe with a barrel capacity of at least 10 mL to confirm patency.

## 3 Treatment strategy

**Figure 2:**
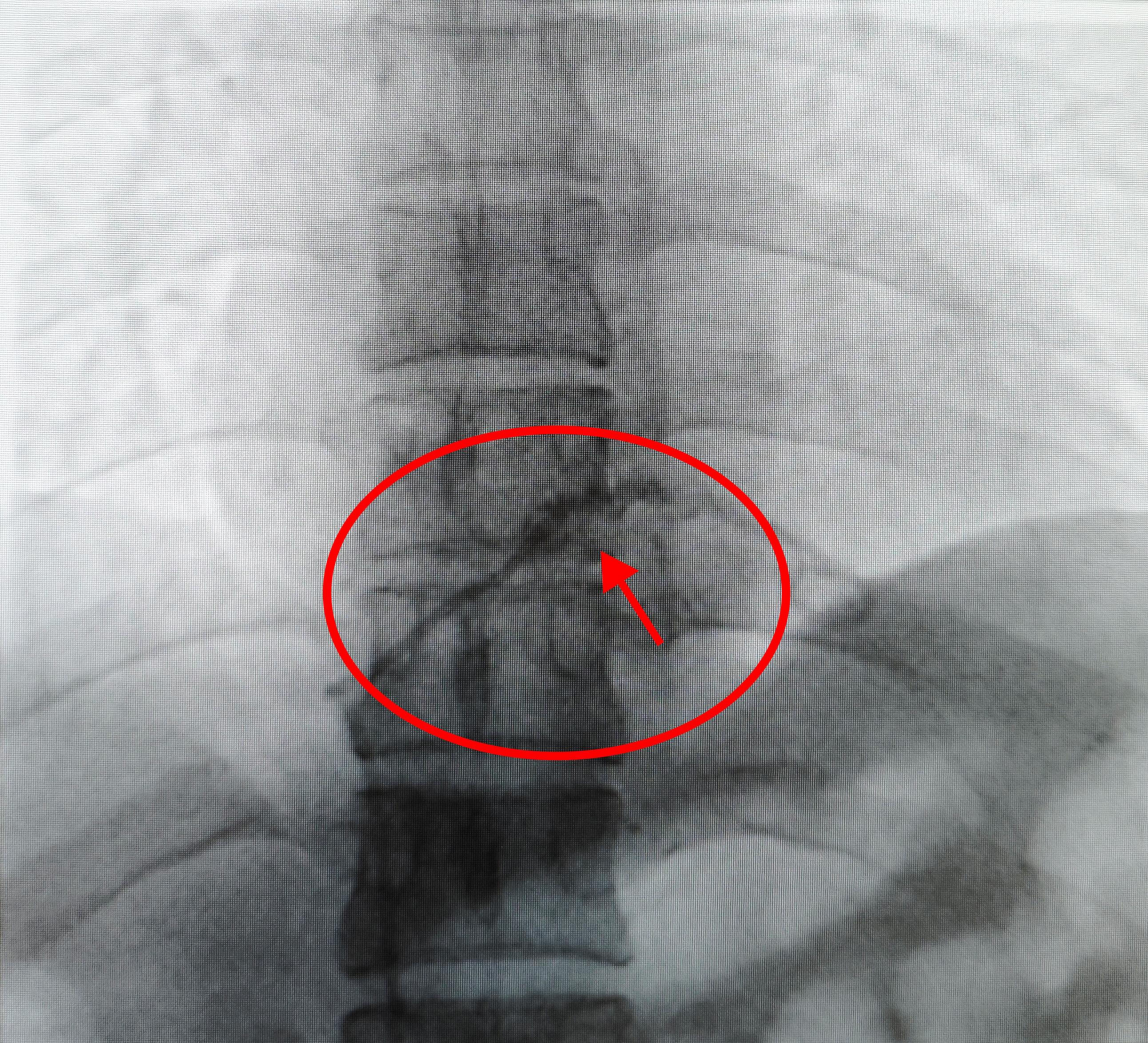
The chest CT report outside the hospital showed that after PICC placement, one of the PICC tips was located in the pulmonary artery corresponding to the dorsal segment of the left lower lobe; and the right atrium and right ventricle appeared to have the shadow of the winding pipe, and the superior vena cava was not clearly visible.

On December 16, 2022, the patient underwent routine PICC maintenance in her local hospital. The local hospital informed that the catheter was unobstructed, but blood could not be drawn out and it was recommended to go to a superior hospital for treatment. On December 17, 2022, the patient went to a local superior hospital for PICC maintenance again. When the medical staff who performed the maintenance opened the catheter dressing, the PICC catheter connector and the exposed catheter had all fallen out. An immediate chest CT examination showed that the PICC catheter was broken. The patient was then admitted to our hospital from the emergency department. When the patient came, the arm on the original catheter side (right upper arm) was not covered with any gauze or transparent gauze. The PICC puncture point was visible to the naked eye, and no bleeding or exudation was seen. No complaints of coughing, chest tightness, shortness of breath, or other discomfort were reported. She was advised to rest in bed and avoid activities. The chest CT report outside the hospital showed that after PICC placement, one of the PICC tips was located in the pulmonary artery corresponding to the dorsal segment of the left lower lobe; and the right atrium and right ventricle appeared to have the shadow of the winding pipe, and the superior vena cava was not clearly visible (**Figure 2**).

**Figure 3:**
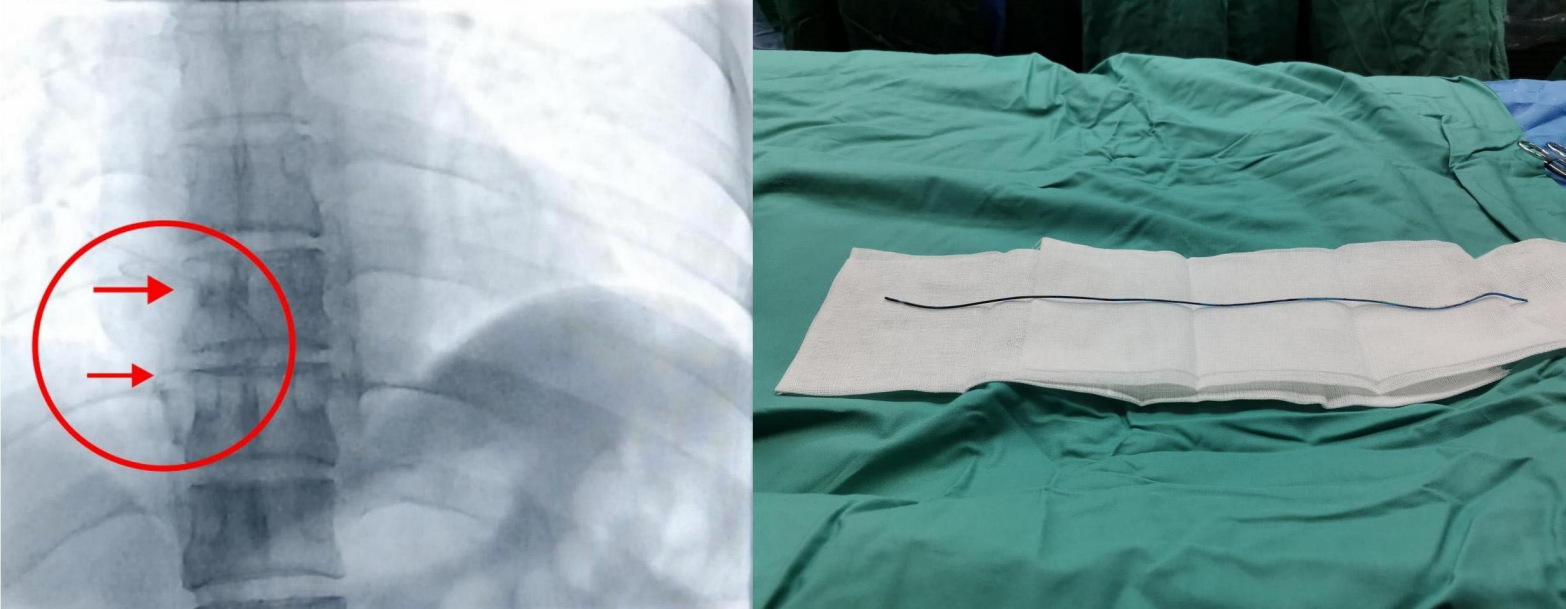
The catheter fragment of 35 cm was removed under direct vision, and then the catheter and vascular sheath were pulled out in turn and sutured layer by layer.

After multidisciplinary discussion, an operation for PICC catheter removal under DSA was about to perform on this patient. Given the patient’s stability, intervention was scheduled electively. For procedural preparation, bilateral groin skin was sterilized as appropriate, and all necessary preoperative assessments—including complete blood count, coagulation studies, infectious screening, and other relevant laboratory tests—were completed to ensure safe conditions for the upcoming intervention. The patient underwent removal of the PICC fragment under DSA under local anesthesia with the supine position. Under fluoroscopy, a PICC fragment was seen floating in the left pulmonary artery trunk at the distal end of the right ventricle. After the routine skin preparation and draping, 2% lidocaine was used for local anesthesia, and the right femoral vein was punctured and a 5F pigtail catheter was successfully introduced. Under the guidance of the black loach guidewire, the pigtail catheter was introduced and rotated clockwise to wrap around the broken tube, and then dragged down to the right groin area under fluoroscopy. After the proximal end was compressed to prevent the catheter from falling off, the skin and subcutaneous tissue were separated layer by layer until the femoral vein was fully exposed. The catheter fragment of 35 cm was removed under direct vision, and then the catheter and vascular sheath were pulled out in turn and sutured layer by layer (**Figure 3**). The operation was successful and there was no discomfort during the operation. After the operation, the anastomosis was protected with sterile gauze, and the patient returned to the ward safely. After the patient was discharged from the hospital after operation, telephone follow-ups were conducted several times and the patient recovered well without any complications.

## 4 Discussion

In clinical practice, the use of PICC can lead to a variety of complications, including catheter occlusion, leakage, malposition, catheter-associated infections, and venous thrombosis (11). Moreover, the PICC may rupture left in place for a long time due to a variety of factors, which further increases the risk of treatment for patients (12). At present, silicone PICCs are increasingly recognized for their fragility and susceptibility to fracture. Current guidelines recommend power-injectable polyurethane PICCs to mitigate this risk (1, 13). In this case, the silicone catheter material was the primary factor in the fracture due to long-term wear between the silicone catheter and stainless-steel connector. In 2013, a 147-center survey indicated that the proportion of silicone material used in PICCs in China’s tertiary hospitals was 72.8% (14). Since 2016, polyurethane catheters have been more widely adopted in China. Due to the lack of the latest data on different PICC material application in China, new surveys are urgently needed. However, silicone catheters still hold a significant market share due to restrictions on China’s medical insurance policies (15). The medical-grade silicone catheter’s connector is stainless steel, so long-term contact and wear between these two parts increase the risk of catheter breakage. This is the objective factor for PICC rupture. While silicone exhibits limited durability and susceptibility to damage, studies indicates that various chemotherapy solutions do not significantly degrade polyurethane or silicone (16, 17). However, prolonged implantation reveals increasingly divergent mechanical performance between these materials. Polyurethane retains its mechanical integrity over time, whereas silicone demonstrates growing heterogeneity in mechanical testing. Consequently, many Western institutions have discontinued silicone catheters in favor of advanced polyurethane PICCs. Clinical evidence confirms that modern polyurethane PICCs offer superior biocompatibility, higher tensile strength, enhanced flexibility post-insertion, and greater chemical resistance. Therefore, discontinuing silicone catheter use represents the most effective strategy for minimizing PICC fractures. However, in this study, we discovered and summarized certain subjective factors in order to provide experience for reducing the risk of PICC catheter rupture in future clinical treatment.

Improper maintenance by nursing staff is a significant contributor to PICC-related complications (18). In our case, inadequate training resources in primary care settings may limit standardized PICC maintenance. The use of excessive force during catheter flushing, for example, can heighten the risk of catheter fracture. Additionally, many hospitals have not fully implemented PICC care protocols, leading to a shortage of professionally trained maintenance personnel (19). Inadequate training leaves some nurses unable to accurately assess the condition of the catheter or the punctured vessel, contributing further to the risk of catheter breakage, especially when care is provided outside of specialized centers. In the future, the standardized care bundle of PICC (i.e. develop a strict nursing plan during the PICC implantation, maintenance and regular monitoring process) should be promoted to hospitals of all levels. Patient-related factors should focus on education regarding activity restrictions to reduce tension on the catheter (20). Moreover, while PICC lines are theoretically designed to remain *in situ* for up to a year, factors such as repeated or excessive bending of the catheter and its extension tubing, improper fixation, and incorrect positioning can predispose the device to bending and eventual wear of the catheter wall over time (12). These issues ultimately elevate the risk of catheter fracture.

Since catheter rupture may be asymptomatic or may cause arrhythmia, timely identification of the emergency situation of rupture is a necessary prerequisite for timely removal of the catheter, which relies on rigorous regular inspections and professional nursing staff (21). In our case, due to the urgency of the patient’s condition, both the patient and her family experienced significant anxiety and distress. The doctors and nursing staff responded with empathy and patience, thoroughly addressing the patient’s concerns and providing reassurance, which helped to ease her emotional state. They emphasized the advantages of interventional procedures, highlighting benefits such as minimal invasiveness and faster recovery, thereby presenting the approach as an effective solution to the clinical need. In addition, the team underscored the technical expertise of the interventional physicians, which helped build further trust with the patient and her family. Continuous collaboration between nurses and physicians ensured open communication with the patient and her relatives: possible complications and corresponding management strategies were fully explained, encouraging patient cooperation and readiness for timely intervention.

Traditional surgical incision to remove foreign bodies in the body, especially foreign bodies in the heart cavity, is complicated, has high surgical risks, and many complications, which some patients may not be able to bear. In this case, the patient underwent PICC retrieval under DSA, which has less trauma, shorter operation time, convenient and simple postoperative care, mild intraoperative and postoperative complications, less impact on the patient’s systemic system, faster postoperative recovery, and reduced pain for the patient, providing an effective method for the treatment of PICC catheter rupture. Additionally, risks associated with DSA-guided catheter removal, such as vascular injury, thromboembolic complications, radiation-related risks, and contrast-induced nephropathy, need to be paid more attention (18). As long as professional medical staff discover and manage it in time, it will not cause harm to the patient. In addition to the satisfaction of patients and their families, it also reflects the value and significance of our specialist nurses, and even more so reflects the high-quality care and professionalism of clinical nurses, which is worthy of promotion in clinical practice. How to prevent the rupture of PICC catheters, reduce medical risks, and ensure medical safety are issues that we should think deeply about in our future work.

## 5 Conclusion

This case highlights the rare but serious complication of PICC catheter fracture with subsequent migration into the pulmonary artery during chemotherapy in a patient with lung adenocarcinoma. Prompt identification and multidisciplinary management, including minimally invasive retrieval under DSA guidance, ensured a favorable outcome and avoided major morbidity. The experience underscores the importance of standardized PICC maintenance protocols, comprehensive training of nursing staff, and patient education on self-care to minimize preventable complications. Furthermore, our findings emphasize the need for regular catheter monitoring and timely intervention when abnormalities are detected. Minimally invasive endovascular techniques represent a well-documented, safe and effective alternative to traditional surgical methods for the retrieval of intravascular foreign bodies, with advantages of reduced trauma, faster recovery, and fewer complications. Continued efforts to enhance nursing skills, strengthen follow-up, adopt polyurethane catheters instead of silicone PICC per current guidelines, and promote patient awareness are essential to improve the safety and success rates of PICC utilization in cancer populations requiring long-term intravenous access.

## Data Availability

The original contributions presented in the study are included in the article/supplementary material. Further inquiries can be directed to the corresponding authors.
